# Clinical implication of bactibilia in moderate to severe acute cholecystitis undergone cholecystostomy following cholecystectomy

**DOI:** 10.1038/s41598-021-91261-9

**Published:** 2021-06-04

**Authors:** Je Ho Yoon, Kwang Yeol Paik, Hoo Young Chung, Ji Seon Oh

**Affiliations:** 1 Department of Surgery, Yeoiudo St. Mary’s Hospital, The Catholic University of Korea College of Medicine, #10,63-ro,Yeongdengpo-gu, Seoul, 07345 South Korea; 2 Department of Family Medicine, Yeoiudo St. Mary’s Hospital, The Catholic University of Korea, #10,63-ro,Yeongdengpo-gu, Seoul, 07345 South Korea

**Keywords:** Gastroenterology, Health care, Medical research

## Abstract

There is little evidence of clinical outcome in using antibiotics during the perioperative phase of acute cholecystitis with bactibilia. The aim of current study is to examine the effect of bactibilia on patients with acute cholecystitis and their perioperative clinical outcome. We performed a retrospective cohort analysis of 128 patients who underwent cholecystectomy for acute cholecystitis with moderate and severe grade. Patients who were positive for bactibilia were compared to bactibilia-negative group in following categories: morbidity, duration of antimicrobial agent use, in-hospital course, and readmission rate. There was no difference in morbidity when patients with bactibilia (n = 70) were compared to those without (n = 58) after cholecystectomy. The duration of antibiotics use and clinical course were also similar in both groups. In severe grade AC group (n = 18), patients used antibiotics and were hospitalized for a significantly longer period of time than those in the moderate grade AC group. The morbidity including surgical site infection, and readmission rates were not significantly different in moderate and severe grade AC groups. In moderate and severe AC groups, bactibilia itself did not predict more complication and worse clinical course. Antibiotics may be safely discontinued within few days after cholecystectomy irrespective of bactibilia when cholecystectomy is successful.

## Introduction

The course and outcome of laparoscopic cholecystectomy (LC) is significantly affected by the severity of inflammation. It is known that prophylactic antibiotics do not prevent infections in patients with mild grade acute cholecystitis (AC) undergoing laparoscopic cholecystectomy^1^. In terms of using postoperative antibiotics, with the intent to reduce subsequent infection sources, rationale for this includes the finding that bacteria in gallbladder (GB) bile is cultured in 40% to 60% of cases^2,3^. In moderate to severe grade AC, clinical course of patients sometimes can be unstable due to the septic condition and it may correlate directly with microorganism in GB, which prolong the use of antibiotics. Nevertheless, the clinical implication of bactibilia remains undefined with the scarcity of scientific evidence. When mild AC is managed with cholecystectomy and the source of infection is controlled completely, prolonged postoperative antibiotic therapy is not warranted^4^. In patients with severe AC, there is no consensus for the use of antibiotics in the postoperative phase of AC. The optimal duration of antibiotic therapy following percutaneous cholecystostomy (PC) is also unknown for PC operated in patients with high risk AC^5^. The controversy remains over whether bactibilia induced by biliary drainage is associated with postoperative infectious complication. Overall, there is little evidence for the use of antibiotics and surgical outcomes during the perioperative phase of AC with regards to bactibilia. We hypothesize that there is difference regarding the use of antibiotics and the surgical outcomes during the perioperative phase of AC with regards to bactibilia. The aim of current study is to examine the association between bactibilia and the clinical outcome of cholecystectomy in different grades of AC.

## Methods

### Patients inclusion criteria

High-risk patients were identified based on the following inclusion criteria: admission into General Surgery for the treatment of AC; American Society of Anesthesiology (ASA) Grade ≥ III and Grade II and III AC according to Tokyo guideline 18 (TG18)^6^. Severe AC (grade III) was defined as being accompanied by dysfunctions in any one of the following organs or systems: cardiovascular dysfunction (hypotension requiring treatment with dopamine > 5 μg/kg per min, or any dose of dobutamine), neurological dysfunction (decreased level of consciousness), respiratory dysfunction (PaO_2_:FIO_2_ratio < 300), renal dysfunction (oliguria, creatinine > 2.0 mg/dL [to convert to μmol/L, multiply by 88.4]), hepatic dysfunction (> 3 prothrombin time: international normalized ratio > 2) or hematologic dysfunction (platelet count < 100,000/μL). Moderate acute calculous cholecystitis (grade II) is defined as being accompanied by any of the following conditions: white blood cell count greater than 18,000/μL, a palpable tender mass in the right upper abdominal quadrant, duration of complaints for more than 72 h, or marked local inflammation (gangrenous cholecystitis, pericholecystic abscess, hepatic abscess, biliary peritonitis, or emphysematous cholecystitis). All LC procedures were performed by a single surgeon (American position and three port method), specialized in performing hepatobiliary procedures. The Institutional Review Board of Yeouido St.Mary’s Hospital approved the current study. A written informed patient consent was waived by the Catholic University, Yeouido St.Mary’s Hospital Institutional Review Board due to the retrospective nature of the study. All methods were conducted in accordance with relevant guidelines and regulations.

### PC and LC

All patients underwent PC in preparation for safe cholecystectomy and for bile culture. PC was performed via transhepatic route. Ultrasound-guided GB puncture was performed using an 18-gauge needle. After a successful puncture, a 0.035-in. wire was inserted, and the needle was removed. Finally, an 8.5-Fr pig tail catheter was inserted into the GB, using the Seldinger technique. All procedures were performed by our specialized interventional radiologist team. Considerations for discharge included stable clinical condition, tolerable pain (using oral analgesics as needed) and proper resumption of oral diet. We performed LC when symptom and sign derived from underlying disease and septic condition were completely improved irrespective of hospitalization.

### Bacteria evaluation and antibiotic treatment

As a surveillance bile culture, bile specimens were collected at the time of PC procedure. All patients received appropriate antibiotic therapy until cholecystectomy and the treatment was discontinued after surgery within a few days. As soon as causative organisms had been identified, antibiotic therapy was adjusted to a narrower spectrum antimicrobial agent based on the specific micro-organism(s) and the results of sensitivity testing. However, antimicrobial therapy could be discontinued prior to identifying the causative organism because it requires several days for bacterial culture to be determined in practical field. Second or third generation cephalosporin were used as initial antibiotic therapy for all patients irrespective of bile culture from PC. The subsequent choice of antibiotics depended on incubated micro-organism and the presence of drug resistance.

### Endpoint

Outcomes used for analysis included demographics, clinical data such as ASA grade, TG18 grade, preoperative bile drainage or endoscopic intervention and prior surgical history. The primary endpoint was occurrence of morbidity which means surgical or medical complications including surgical site infection (SSI). The secondary endpoints were the duration of antibiotic usage pre- and post-operation, hospitalization period, postoperative stay (from the time of the index surgery to discharge), and the rate of readmission (admission after cholecystectomy).

### Statistical analysis

Statistical analysis was performed using SPSS software (version 24.0; IBM SPSS Statistics, Armonk, NY, USA). Student’s t-test or Pearson’s chi-squared test and Fisher’s exact test were used for between-group comparisons, as appropriate for the data type and distribution. For all analyses, a P-value < 0.05 was considered as statistically significant.

## Results

### Patients

From January 2014 to December 2018, 455 patients with AC underwent LC in the Department of Surgery at the xxx University of Korea, xx Hospital. Among them, 164 high-risk patients who were initially intended for cholecystectomy underwent PC. Of 164 patients, 8 patients who could not receive surgery due to their clinical state and 4 patients who underwent open cholecystectomy were excluded. Then, 24 patients without culture report (21.5%) were also excluded*.* Finally, 128 patients were enrolled in the study. Patients were divided into two groups depending on the presence of bactibilia and to the severity grade as moderate and severe grade according to the TG18.

### Microorganism

Out of 128 patients with AC who underwent LC, there were 70 (54.7%) bactibilia patients and 58 (45.3%) bile culture-negative patients**.** Thirty bacteria were identified in 70 patients and 36 (51.4%) patients were infected by single gram negative bacilli (GNB) and 10 patients showed concomitantly infected multi-GNB. Of all GNB, *Escherichia coli* was the most frequently isolated bacteria (n = 24), followed by *Klebsiella pneumonia* (n = 8) and Enterobacter (n = 6). Gram positive cocci (GPC) occupied 27.1% (n = 19) of all patients with bactibilia and most of them were Enterococci (n = 6). *Pseudomonas aeruginosa*, anaerobes (Corynebacterium) were isolated in 7.2% (n = 5) of patients. Thirteen patients had GNB and GBC concomitantly. The bile culture results are presented in Table 1.

**Table 1:** Bacterial growth via percutaneous cholecystostomy.

	N = 70
**Gram positive cocci (GPC)**	
*Enterococcus faecium*	6 (8.6%)
*Enterococcus faecalis*	
*Streptococcus viridans*	
*Enterococcus durans*	
**Gram negative bacilli (GNB)**	
*Escherichia coli*	36 (51.4%)
*Klebsiella pneumoniae*	
*Klebsiella oxytoca*	
*Klebsiella aerogenes*	
*Aeromonas hydrophila group*	
*Citrobacter braakii*	
*Enterobacter cloacae*	
**GNB multiple**	
*Escherichia coli*	10 (14.3%)
*Klebsiella pneumoniae*	
*Morganella morganii*	
*Klebsiella oxytoca*	
*Proteus vulgaris*	
**GPC + GNB**	
*Escherichia coli*	13 (18.6%)
*Escherichia coli*	
*Klebsiella pneumoniae*	
*Klebsiella oxytoca*	
*Klebsiella aerogenes*	
*Aeromonas hydrophila group*	
*Citrobacter braakii*	
*Enterobacter cloacae*	
*Raoultella planticola*	
*Enterococcus gallinarum*	
*Enterococcus casseliflavus*	
**Others**	
*Corynebacterium* sp.	5 (7.2%)
*Pseudomonas aeruginosa*	

### Perioperative outcome according to the bactibilia

The clinical results of the bactibilia and the culture-negative groups are presented in Table 2. The age of patients that comprise the bactibilia group is older than patients in the culture-negative group (68.5 vs 63.9 years, p < 0.001). The proportion of patients with ASA grade III is 12.9% in the bactibilia group and 15.5% in the non-culture group (p = 0.536), and the proportion of severe grade AC is 8.6% in the bactibilia and 20.7% in the control group. However, there was no significant difference in both groups (p = 0.073). Furthermore, the duration of antibiotics use and the presence of bactibilia (total: 7.4 days vs 8.2 days, p = 0.401 and preoperative: 5.8 days vs 6.9 days) was not significantly correlated. Also, readmission rate (7.1% vs 3.4%) and overall morbidity (8.6% vs 8.6%) were not different in both groups. Incisional and organ/space surgical site infection (SSI) occurred in 2 (2.9%) patients with bactibilia and none in culture-negative group respectively (p = 0.297). Mulitivariate analysis was not performed because bactibilia did not affect morbidity and clinical course in univariate analysis.

**Table 2:** Perioperative outcome according to bactibilia.

	Bactibilia (n = 70)	None (n = 58)	p-value
Age	68.5 ± 9.9	63.9 ± 15.6	0.000
Gender (male)	50 (71.4%)	35 (60.3%)	0.195
**Tokyo guideline**			0.073
Severity II	64 (91.4%)	46 (79.3%)	
Severity III	6 (8.6%)	12 (20.7%)	
**ASA**			0.536
I	4 (5.7%)	6 (10.3%)	
II	57 (81.4%)	43 (74.1%)	
III	9 (12.9%)	9 (15.5%)	
Total bilirubin	2.0 ± 1.6	1.9 ± 18	0.356
ERCP	6 (8.6%)	4 (6.9%)	0.495
Preopeartive bile drainge	3 (4.3%)	0 (0)	0.251
PC- operation day	5.8 ± 4.0	6.9 ± 4.9	0.593
Morbidity	6 (8.6%)	5 (8.6%)	0.617
SSI	2 (2.9%)	0 (0)	0.297
Readmission	5 (7.1%)	2 (3.4%)	0.455
Postoperative day	3.7 ± 2.9	3.4 ± 2.7	0.526
Per oral anti day	1.5 ± 2.6	1.3 ± 2.7	0.706
Total antibiotics day	7.4 ± 4.6	8.2 ± 0.2	0.401

### Perioperative outcomes according to the severity of acute calculous cholecystitis

For the severe grade AC patients, preoperative duration of antibiotic use was 9 days, which was longer than 5.9 days of culture negative group (p = 0.006). The overall duration of antibiotic use was also longer (11.9 days vs 7.8 days, p < 0.001) in the severe grade AC patients. Postoperative hospitalization of patients with severe grade was also longer than that of moderate grade patients (5.4 days va 3.3 days, p = 0.003). The postoperative SSI, morbidity, readmission rates did not differ between the two groups. Unexpectedly, bactibilia was more common in the moderate group (58.2%) than in the severe group (33.3%). The clinical results according to AC severity were presented in Table 3.

**Table 3:** Perioperative outcome according to disease severity.

	Moderate (n = 110)	Severe (n = 18)	p-value
Age	65.9 ± 13.2	69.8 ± 11.2	0.237
Gender	70 (63.6%)	15 (83.3%)	0.081
**ASA**			0.269
I/ II/ III	10 /86/14	0/ 14/ 4	
ERCP	9 (8.2%)	1 (5.6%)	0.575
Total bilirubin	1.9 ± 1.5	2.6 ± 2.3	0.101
Preopeartive bile drainge	10 (9.1%)	1 (5.6%)	0.522
Bactibilia	64 (58.2%)	6 (33.3%)	0.044
PC-LC day	5.9 ± 4.1	9.0 ± 6.2	0.006
Per oral anti day	1.2 ± 2.1	2.9 ± 4.6	0.008
Postoperative day	3.3 ± 2.4	5.4 ± 4.5	0.003
Total antibiotics day	7.8 ± 4.3	11.9 ± 8.8	0.000
SSI	1 (0.9%)	1 (5.6%)	0.264
Morbidity	3 (2.7%)	2 (11.1%)	0.145
Readmission	6 (5.5%)	1 (5.6%)	0.663

## Discussion

Despite bactibilia being a common finding in AC and a potential risk factor for worse clinical course, the present study found no significant correlation between bactibilia and negative postoperative outcomes of cholecystectomy. However, the study demonstrated that the severe grade AC is associated with a longer use of perioperative antibiotics and prolonged hospitalization. For the underlying impact of bactibilia, we excluded mild grade cholecystitis in this study and only included moderate and severe grade inflammation.

The prevalence of bactibilia is significantly higher in moderate grade AC patients than in mild grade AC patients^6^. Additionally, patients with uncomplicated cholelithiasis have aseptic bile^7^. Tokyo guidelines recommend that bile culture should be performed at all available opportunities, especially in severe cases^6^. In terms of mild to moderate grade ACs, recent RCT revealed that patients who received preoperative and intraoperative antibiotics, despite the fact that they did not receive postoperative treatment with amoxicillin plus clavulanic acid, did not result in a greater incidence of postoperative infections^4^. Regarding moderate to severe grade ACs, it is not conclusive whether clinical implication of bactibilia is associated with poorer clinical outcome pre- and post-operation. Overall, there were low level of evidence regarding the correlation of bactibilia and poor clinical outcome such as surgical complication. Some authors revealed that bactibilia is a significant factor associated with infectious complications and prolonged hospitalization after surgery^6,8–10^. However, majority of them did not separate the grade of inflammation as a possible confounding factor. These studies proved a trend towards increased incidence of positive bile cultures in the prolonged antibiotic group, which may have affected decisions regarding antibiotic therapy^3,8,10^. In this study, there was no correlation of surgical complication and bactibila. Incisional and organ/space SSI occurred in only two patients with bactibilia and none in culture-negative group respectively. Readmission rate and overall morbidity was not different in the bactibilia group and the culture-negative group. Consequently, there was no significant correlation between the preoperative and overall duration of antibiotics use and bactibilia.

In obstructive jaundice induced by periampullary neoplasm preoperative, surveillance bile culture is useful for the management of wound infection and prediction of causative pathogens for infectious complications^11^. The incidence of infectious complications is higher when performing cholecystectomy after ERCP^12^. In our series, the number of patients who underwent preoperative ERCP was only 7.8%. Thus, the influence of bactibilia in cholangitis may be minimal compared to other studies. In the present study, bactibilia was revealed to occur at a frequency of 54.6% in overall patients; 58% in moderate and 33.3% in severe inflammation. Yoon et al.^12^ showed 25% of bactibilia in their study including only mild grade. Overall, our study may present more precise implication regarding bactibilia with its negative results. There is no consensus for the use of antibiotics in the postoperative phase of severe AC. There has been no report up to date of detailed bacteriological analysis of bile in patients with AC graded in severity according to the Tokyo guidelines. Most of the patients were classified into mild and moderate severity; severe cases were extremely rare^6^.

We analyzed bactibilia for AC patients according to the Tokyo guidelines. For the severe grade AC patients, preoperative duration of antibiotic use was 9 days, which is much longer than 5.9 days of culture in the bactibilia negative group and the overall duration of antibiotic use was also longer. Despite such difference, the postoperative SSI, morbidity, readmission rates were not significantly different in these groups. Comparison of moderate and severe grades revealed that prolonged hospitalization is associated with severe grade of AC without increasing postoperative complication. Considering our results, routine bile culture may not be recommended in patients undergoing PC especially following cholecystectomy.

In general, empirically selected broad-spectrum antibiotic therapy should be prescribed according to the severity of cholecystitis. As soon as causative organisms are identified, antibiotic therapy should be adjusted to a narrower spectrum antimicrobial agent based on the specific micro-organism and the results of sensitivity testing. However, antimicrobial therapy usually deceased prior to identify causative organism needs several days after culture in practical field. Especially recovery period after cholecystectomy is relatively short compared to other major surgeries, traditionally long-term antibiotic therapy after cholecystectomy is not required irrespective of inflammation grades. Therefore, precise duration of antibiotics cannot be presented in this study. Because we did perform PC in all patients, the mean duration of antibiotic use prior to cholecystectomy was 7 days. We did not follow the recommendation regarding the importance of the timing of the cholecystectomy, which is no longer only the golden 72 h, but perform as soon as possible^13–15^. Other limitation is that we found that there was a trend towards bactibilia being associated with lesser severe ACs. The percentage of bactibilia found in severe grade AC group was 8.6%, while 20.7% of patients had bactibilita in moderate AC group. Overall, our data indicate that decisions regarding the antibiotic therapy following PC for acute cholecystitis are arbitrary and that prolonged courses of antibiotics provide no benefit.

In conclusion, bactibilia, in general, does not increase the risk of developing a postoperative complication following cholecystostomy and the presence of bacteria in GB cultures does not correlate with the development of complication after cholecystectomy. Overall, in moderate and severe cholecystitis, bactibilia itself does not predict more complication and worse clinical course. Antibiotics may be safely discontinued within few days after cholecystectomy irrespective of bactibilia when cholecystectomy is successful.
